# Emphasise capability, not disability: exploring public perceptions, facilitators and barriers to living well with dementia in Northern Ireland

**DOI:** 10.1186/s12877-020-01933-w

**Published:** 2020-12-03

**Authors:** Gary Mitchell, Victoria McTurk, Gillian Carter, Christine Brown-Wilson

**Affiliations:** School of Nursing and Midwifery, Medical Biology Centre, Queen’s University Belfast, 97 Lisburn Road, County Antrim, Northern Ireland BT9 7BL

**Keywords:** Dementia, Dementia-friendly, Dementia perception, Person-Centred, Qualitative research, Public health

## Abstract

**Background:**

Improving public knowledge and understanding about dementia has been identified as a priority area by people living with the condition, researchers, educators, and policymakers for several years. Societies that have a better understanding of the condition are more likely to enable people living with dementia to enjoy a better quality of life. The aim of this study was to explore current public perceptions of dementia along with the facilitators and barriers to living well from the perspective of people living with the condition in Northern Ireland.

**Methods:**

Four focus group interviews were conducted with a total of 20 people living with dementia across three Northern Irish Counties in June 2019. These interviews were audio-recorded, transcribed verbatim and analysed using thematic analysis. Ethical approval was obtained for this study prior to data collection.

**Findings:**

Following thematic analysis, three themes emerged in relation to barriers and facilitators to living well with dementia. These were: ‘Emphasis on Disability NOT Capability’, which highlighted societal misconceptions about the activities and modes of life which people with dementia could or could not do; ‘Normalise Dementia – We Don’t Want a Fool’s Pardon’, which focused on how the public could encourage people living with the condition to enjoy greater independence, and ‘Dementia isn’t a Death Sentence’, which considered how professionals, family members and friends treated the person after diagnosis.

**Conclusions:**

Public perceptions about dementia have the potential to act as both facilitators and barriers to living well with dementia. People with dementia stated that they are more likely sustain wellbeing when they are valued and can maintain independence. On the contrary, poor public and professional attitudes to dementia had the potential to disempower people living with dementia.

## Introduction

More than 850,000 people live with dementia in the United Kingdom and this number is expected to surpass one million in the next 5 years [1]. Northern Ireland is part of the United Kingdom, which is projected to have the largest increase, with the number of people with dementia rising by 95% over the next 20 years [2]. This increasing prevalence has led to a greater public awareness of the condition and an increasing number of dementia-friendly communities [3]. These communities have the goal of reducing stigma, promoting greater public understanding of dementia, and ensuring communities actively facilitate people with dementia to make decisions about their lives [4]. While these improvements in public perception about dementia are likely to support people with the condition to live well in their community, people living with dementia may still face challenges [5–7].

The challenges people with dementia may face can manifest in a variety of ways. Arguably the biggest misconception the public have about dementia is that the disease is a normal part of the ageing process [8]. This misconception can prohibit timely diagnosis of dementia and have implications for commencement of treatment [9]. Another challenge commonly reported by people living with dementia is the language used by the media and the public in describing dementia [10]. Offensive or labelling language, such as referring to people living with dementia as a ‘sufferer’ or ‘demented’ perpetuates the stigma associated with the disease and can disempower people living with dementia and their ability to maintain independence [11–13]. The Capabilities Approach, developed by Nussbaum [14], emphasises the promotion of wellbeing through enabling individuals to realise their capabilities and to engage in behaviours they value, therefore focusing on what people can be and do in their lives [15, 16].This Capabilities Approach has been adapted for use in dementia through the Capabilities Model of Dementia Care (CMDC) [17, 18] and this can help to identify barriers such as stigma which can impede opportunities for people to live well with dementia.

There have been several international research studies which have sought to explore public perceptions of dementia and all have concluded that interventions to improve public knowledge about dementia are a priority [19–21]. If public knowledge and perceptions of the condition are limited, people living with dementia are likely to experience disempowerment [21]. Additionally, it is important that family carers are not overlooked for the knowledge they have developed in their role and are supported in care planning decisions [21].

The aim of this study was to explore current public perceptions of dementia along with the facilitators and barriers to living well from the perspective of people living with the condition in Northern Ireland. The reported study findings have been used to inform another project being led by the authors; the co-development of a digital game to improve public perception of dementia: www.dementiagame.com

## Methods

### Design

An interpretive qualitative approach was used based on the belief that social reality is shaped by both human experience and social context [22]. Therefore, this study sought to elicit how people living with dementia understood the public perception of dementia within their everyday experience. Data were collected using focus groups enabling people living with dementia to describe their everyday experience as well as commenting on the experience of others. Using multiple focus groups has the potential for researchers to identify the similarities and differences of experience within and across groups [23].

### Ethical considerations and recruitment

This study received ethical approval by Queen’s University Belfast, School of Nursing and Midwifery Research Ethics Committee in April 2019 (Reference: CBrownWilson 03.19 M2.V1).

Dementia NI, a voluntary membership organisation led by individuals living with dementia, acted as a gatekeeper for this study (https://www.dementiani.org/). Dementia NI have several local empowerment groups that are comprised solely of people living with dementia and their overall function is to contribute to improving services for people with dementia. Dementia NI shared information about the study with members during local empowerment group meetings. People with dementia who were interested in participating in this study attended a focus group meeting 2 weeks later. Immediately prior to the focus group, all participants met with the researcher (GM) who provided information about the study, the structure of the interview and what would happen with the data. The consent processes were guided by Dewing [24] and consent was re-checked at the conclusion of each focus-group. An empowerment officer from Dementia NI, who knew and regularly met with the participants, was present during all periods of data collection to identify if people living with dementia were showing signs of ill being that could imply consent was being withdrawn during the focus group.

### Sample

All participants met the inclusion criteria which included the following: living with a formal diagnosis of dementia and having the cognitive ability to actively participate in discussions about the facilitators and barriers to living well with dementia in their local communities.

### Data collection

A total of 20 people living with dementia participated in four focus group interviews. All participants had received a formal diagnosis of dementia in the past 5 years, lived alone or with family and were aged between 52 and 78. Fourteen participants were female, and six participants were male. Participants lived with a variety of dementias including, Alzheimer’s Disease, Vascular Dementia, Lewy-Body Dementia and Frontotemporal Dementia. All participants were in the early to middle stages of their disease, did not currently require formal care and had capacity to consent to participating in the focus group and engage fully with discussions.

To protect the confidentiality of participants, we are unable to report further specific demographic details of individuals. There were five participants in focus group one, five participants in focus group two, three participants in focus group three and seven participants in focus group four. Family members or friends did not accompany people living with dementia to these focus group, though this choice was available to all participants.

Focus groups took place throughout June 2019 at four different venues in Northern Ireland, representing both rural and urban communities. Locations were selected by Dementia NI. The duration of focus-group interviews was between 35 min and 75 min. All focus group interviews were moderated by one facilitator (GM) who had clinical expertise in dementia and previous focus group interview experience. An interview guide was not used to facilitate focus-group discussions. An unstructured interview was preferable to enable people living with dementia to identify their own perspectives rather than being guided by the interviewer. Each focus group was asked the following open ended questions: ‘*Tell us about a positive everyday experience when you are out and about; what made this experience positive*’, followed by *‘Tell us about a not so positive experience you might have had; what made this experience not so positive’*. This approach to questioning allowed the participants to discuss the facilitators and barriers to living well with dementia in an informal conversational manner. A range of prompts were used when required such as *‘Did this make you feel rushed?* Or *‘Did this give you enough time?*

### Data analysis

Data were transcribed verbatim and analysed using thematic analysis based on Braun and Clark’s [25] six stage framework: familiarisation with data, generating initial codes, searching for themes, reviewing themes, defining themes, and producing the report. Two members of the team (GM & VM) read and re-read the data independently to generate initial codes. Once the initial codes were agreed, these were formed into themes by the same members and reviewed by the other members of the team (CBW & GC). The codes and themes were reviewed by members of the Dementia NI empowerment groups who contributed to defining and finalising the themes. Table 1 provides an example of this coding.

**Table 1:**
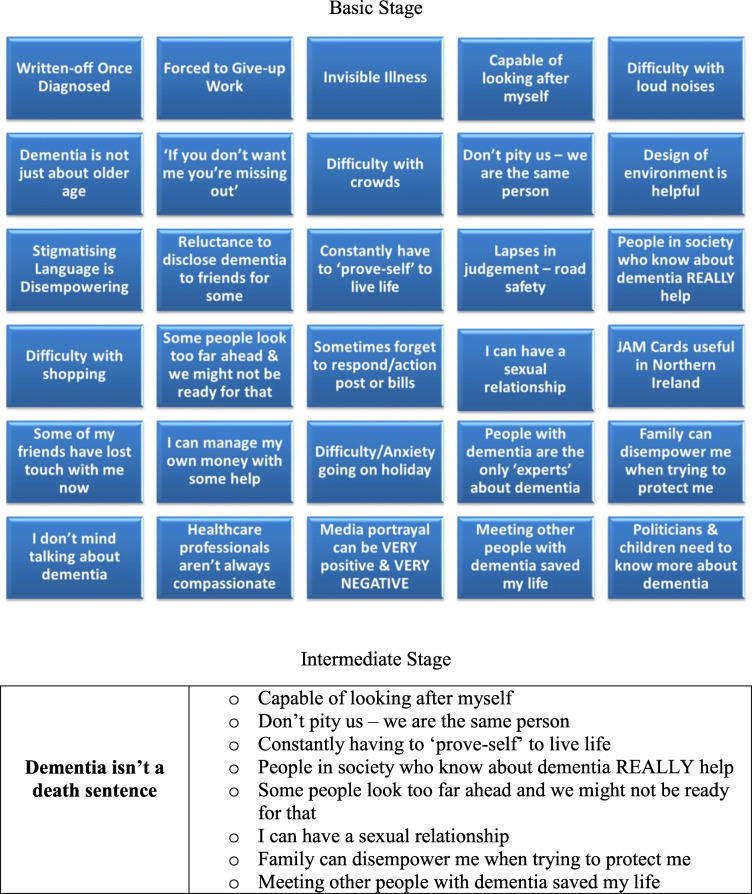
Coding Example

### Trustworthiness

To improve the trustworthiness of research data the four criteria of credibility, transferability, dependability, and confirmability were followed [26]. Participants were provided with an opportunity to review the raw and themed data. Comprehensive record-keeping was undertaken and retained throughout the research process. Regular team meetings ensured an appropriate audit trail was followed and reflexivity was addressed using a reflective diary that was updated after each focus group. The reflexive diary was used as an ongoing check of the interviewer’s experiences, as a log of the data collection activities as they occurred and as a record of the methodological decisions made during the research process [26].

## Results

Following thematic analysis, three themes emerged in relation to barriers and facilitators to living well with dementia. These were: ‘Emphasis on Disability NOT Capability’, ‘Normalise Dementia – We Don’t Want a Fool’s Pardon’ and ‘Dementia isn’t a Death Sentence’.

### Theme one: “often the emphasis is on disability NOT capability”

Throughout the focus group interviews, it was highlighted that people living with dementia appeared to lose a significant amount of their independence once they were formally diagnosed. The participants collectively felt that they were encouraged to rely on their family and friends to do everyday tasks and make decisions, rather than maintain their independence first. People with dementia discussed the importance of decision-making after diagnosis with one participant stating the following:

*‘I make my own decisions, if I want something, I get it and if I don’t…well I won’t. It’s my decision.’*

*FG1P2*

During the focus groups, it was evident that every participant felt strongly about maintaining their independence. The participants would emphasise that, as a person, they had not changed at all. Participants also articulated their capability in relation to normal activities, for example shopping, money management, driving, living alone and socialising with friends. An example of this was highlighted by one lady who had been living with dementia for more than 4 years:

*‘I am still quite capable of looking after myself and my bank account. I can get up in the morning. I can shower, I can dress myself, and I can go out to the shops, meet a friend for coffee… I am quite capable of looking after myself’.*

*FG3P3*

While participants provided lots of examples of their capability, they conveyed a sense of frustration with wider society who they felt often had misconceptions about disabilities perceived to be associated with dementia. All participants were aware that their dementia would inevitably lead to cognitive decline and challenges with maintaining independence, however there was a strong feeling that societal focus is often not about independence. One of the main elements discussed was a person’s ability to drive post-diagnosis. While participants understood the health and safety implications of driving with dementia, many felt dependent on family or friends while they awaited a driving retest to examine their capability which often could take considerable time. This impact prevented many people from carrying on with their everyday life.

Another aspect discussed within these groups was the capability of performing the task of shopping and dealing with money. In terms of shopping, most people living with dementia stated that they often had to shop with a partner/family or friend due to their own concerns about confusion, anxiety or getting lost during busier times. One key challenge which led people with dementia to feel more dependent on family or friends related to the frequently changing location of items in shops.

*‘If I went into a shop to get my groceries, were you know which isle to go to get your stuff and … even in ‘Home Bargains’ [Shop] when you go back into it, they’ve moved the stuff all around’*

*FG4P7*

*‘Just when you’re used to a thing, the next week it’s all moved around’*

*FG1P5*

One local intervention, which participants thought was extremely useful, was the JAM card (https://jamcard.org/). The JAM acronym means ‘just a minute’ and the laminated card has been designed to support people with dementia in telling others that they need ‘Just a Minute’ in a discrete manner. People throughout the four focus-group interviews expressed how the JAM card made life easier, particularly when paying for items at a shop, using public transport or becoming lost in a familiar place.

*‘The JAM card would be used when you’re trying to count out your money out in a shop…and you just show them [the shop keeper] your pass and then they would say ‘oh hold on yes’ and they will give you a bit of time to get yourself organised’.*

*FG3P1*

*‘The JAM Card alone has helped me. It is a little comforter that I don’t always need, but if things are racing a bit, I just take the card out [and show this to the service-provider] and that helps put the brakes on’*

*FG1P3*

The JAM card was a tangible example how local communities could enable people living with dementia to be independent through focusing on their capability, not their disability. People who participated in the focus-groups also stated that people’s attitude was a major facilitator in supporting independence.

### Theme two: “normalise dementia – we don’t want a Fool’s pardon!”

One significant barrier to ‘living well’ was the general public’s interpretation of what dementia is and how they believe it affects people living with the disease. Focus group participants wanted dementia to be normalised and *viewed* as an illness, much like heart failure or cancer. These people living with dementia also wanted the public to be aware of the symptoms of dementia and use this knowledge to help make their life more manageable. One aspect which people with dementia found particularly distressing was the potential of being labelled as a ‘fool’ due to the symptoms of the disease.

While the task of normalising dementia often rested on key members of each person’s local community, like shop-keepers, law enforcement and health services, participants collectively asserted that the media had a huge part to play in positively influencing or informing the views of the wider public. Multiple participants spoke of the positive portrayal of dementia in a recent UK television documentary. The documentary, entitled ‘our dementia choir’ followed the journey of people living with dementia who formed a choir.

*‘I thought it was fantastic! It was nice to see just how they [the public] were reacting, and I mean they [people with dementia] were just normal, you know?’ It was very positive…It’s not something you need to hide’.*

*FG4P1*

People with dementia also discussed the positivity of recent local public health campaigns that had promoted awareness about the condition to the public. In Northern Ireland, the ‘Still Me’ campaign was a media campaign produced by the Public Health Agency to raise awareness of the signs of dementia and to reduce stigma about the condition. People living with dementia felt a sense of empowerment with the campaign as stated below:

*‘It [the campaign] was letting more people know, don’t be scared, if you’ve got dementia don’t be scared.’*

*FG2P4*

While participants were keen to describe the positive contributions that the media could make to improving public awareness of dementia, many also highlighted the negative media portrayals. They felt this exacerbated stigma associated with dementia and disempowered participants. Participants consistently noted obstructive language used, in particular the use of labels such as *‘sufferer’, ‘demented’, ‘crazy’, ‘not-all-there’* and *‘child-like’* were extremely upsetting.

Focus-group participants also pointed to local employers as another key group that could do much more to normalise dementia. Several participants in the focus-group stated that the worst thing about being diagnosed with dementia was being forced by their employers to give-up their work.

*‘Losing your job? That’s the biggest thing, that was a really REALLY hard thing to have happen to you…losing work was like losing a limb. You’ve now lost everything’.*

*FG4P4*

Many participants felt that their formal diagnosis of dementia did not immediately change their level of function and often felt surprised when asked to give up work. They conveyed a sense of abandonment and confusion but strongly concluded that having the right to employment, or modification of one’s work role, was a pre-requisite for the normalisation of dementia. One person with dementia provided the following advice regarding employment:

*‘Learn about dementia to start with…that is what I would say and see what causes it and then make a decision as to why you should employ someone or not. Meet the person and actually see what stage of the dementia they are at’*

*FG2P2*

Participants agreed that to normalise dementia the general public needed to see that people living with the condition can enjoy life through work and activity. The participants did acknowledge that the symptoms of dementia were often invisible earlier in the disease and independent living would become more difficult in time. While public support was always advantageous, participants unanimously agreed that never wanted to receive a *‘fool’s pardon’* because they lived with dementia.

### Theme three: “dementia isn’t a death sentence”

The final theme that emerged from the focus-group data was around healthcare professional and family member treatment of people living with dementia post-diagnosis. The participants across all focus-groups expressed their surprise and dissatisfaction about how healthcare professionals’ treatment of people with dementia often lacked understanding or empathy. The participants collectively felt that healthcare professionals perceived dementia as a death sentence.

*‘They [healthcare professionals] really ought to know better, right? I mean…my GP [General Practitioner] has been great, but the people I visit at the memory clinics…well, I am just a number. The information is all about future planning, worsening symptoms and what have you. What I really want to talk about is how I can live life to the fullest!’*

*FG4P1*

These sentiments were echoed by several participants. Collectively, there appeared to be a professional focus on symptoms of dementia and a consistent reinforcement that dementia is a progressive incurable condition. One participant stated that she was advised by healthcare professionals that she should begin to put an advance care plan in place, a technique that is recommended by several clinical guidelines.

*‘I mean for God’s Sake…I had only just been diagnosed with dementia and they were talking about Power of Attorney this and write a Will that…I was so overwhelmed and I was just sat there thinking – how long have I got left [to live]? It took me a few months myself to realise, actually dementia isn’t a death sentence and there is plenty of fun still to be had!’*

*FG1P3*

There were some positive accounts of healthcare professional interactions with participants, but portrayals of their input were often negative. Although the important role their family members and friends played in facilitating and enabling them to live well was widely acknowledged, at times many people with dementia perceived their family members to be, like healthcare professionals, looking too far ahead into the future. One example of this comes from a participant who, after being diagnosed, was taken by her family to a nursing home to have a look around.

*‘Visiting the home…kind of left me a bit stunned. I’m not there yet, my head was shouting at them, but I couldn’t say anything. I know they are trying to help me and wrap me in cotton wool to protect me because they are afraid…but I am ok. I can still go out to the pub and have a drink and a dance. I don’t particularly want to live in a place with people who I don’t know – I don’t need care yet!’*

*FG3P2*

The role of the family member and healthcare professional was important to people living with dementia. These individuals could support people to overcome challenges in society or could behave in a way which inadvertently perpetuates societal stigma of dementia.

## Discussion

The participants in this study have identified several facilitators and barriers to living well with dementia. This study demonstrates that people living with dementia often experience members of the public who believe they are no longer capable of managing their everyday lives. Whilst dementia does place limitations on their lives, there is still much people with dementia can do and have to offer, a fact that society may ignore. These findings are not unique when considering the global evidence-base. For example, most of the participants identified how they had to give up their job as soon as they received a diagnosis of dementia rather than being offered alternative opportunities within their place of employment [27–29]. Other participants spoke about their lives being placed on hold as their abilities to drive were being reviewed [30]. This contributed to negative public assumptions that people living with dementia were simply unable to do things, which was not always the case. These misunderstandings may also have acted as significant barriers to living well with the condition. Participants spoke of the difficulty in overcoming these challenges and how public understanding could either act as a facilitator or barrier to living well. These findings are strongly reflective of existing literature on the topic [2, 3, 21, 31–33]. Within the Northern Irish context, there is evidence to suggest that the public have satisfactory knowledge about dementia, but this does not always translate into positive attitudes about the condition [34]. While participants in this study did acknowledge facilitators to living well, they also provided many examples of stigmatising public attitudes, for example the use of labelling language. Incidentally, these findings resonate with a large Northern Irish survey more than a decade ago [34].

The participants in this study identified the role of the media in promoting the normalisation of dementia and considered how these positive messages might encourage positive changes in attitude. The Dementia Engagement and Empowerment Project (DEEP) is an organisation which have taken an active approach to this issue and have produced guidelines on language about dementia for journalists, organisations, and communication departments [35]. Indeed, the positive public perception of dementia has the potential to support people with the condition to live well in their local communities [36]. In the UK and Northern Ireland, there are more than 200 dementia friendly communities [37]. These communities prioritise local community action in relation to several key areas which include the arts, culture, leisure, business, children, young people, faith groups, housing, health, and social care and transport [37]. Dementia friendly communities are therefore likely to provide an opportunity to both emphasise capability and normalise living with the condition. In the UK, the Alzheimer Society’s ‘Dementia Friends’ awareness campaign has also been a significant component of educating local public about dementia and dispelling myths associated with the condition [38].

Healthcare professionals that people with dementia encounter during their journey play a key role in their experience. In the current study, people living with dementia were shocked and distressed by the focus of health care professionals and subsequently by their families on the end stages of the condition. As potential societal role-models, due to their medical knowledge of the condition, many participants expressed disappointment about how these professionals appeared to prioritise medical needs and focus on long-term planning. Participants articulated that they did not perceive that living with dementia was a death sentence because there was still much to be enjoyed. Medicalised and paternalistic approaches to people with dementia are commonly discussed in the literature. A recent Irish study found that many people living with dementia were excluded from care-planning and decision-making due to assumptions they lacked capacity or because of family member preference [39]. Some participants in this study expressed feelings of occupying a passive role in their relationships with healthcare professionals and, in some cases, family members. Participants expressed preference for approaches which actively involved and empowered them to maximise independence and live well with their condition. There is a plethora of literature to support such approaches [21, 40–42].

Facilitating people to live well with dementia in their local communities requires improvements in public perceptions and meaningful behavioural change. Participants in this study have spoken about the facilitators to living well in their community and the positive impact this had. It appears that participants who can continue to do what they are capable of are probably more likely to experience higher levels of wellbeing. This concept, known as the capabilities approach, developed by Nussbaum [14], asserts that to live well all people must do and be what they value and lead the type of life they are able to lead. More recently this approach has been adapted to people living with dementia, The Capabilities Model of Dementia Care (CMDC), by Moyle et al. [17, 18]. The CMDC provides a useful theoretical underpinning to this research because most positively associated feelings captured in this study related to Moyle et al. [17, 18] ten central human capabilities. This included feeling valued, living independently, enjoying pleasurable experiences, living in a natural way, experiencing a sense of control, and expressing emotion. In this study participants expressed these central human capabilities in relation to driving, employment, managing their own money, meeting friends, and actively participating in decisions about their life. Communities that place an emphasis on providing proactive and supportive environments, focusing on the strengths and capabilities of people with dementia rather than their deficits, are more likely to facilitate opportunities for living well as noted by participants in this study.

### Strengths and limitations

The strength of this paper was that it engaged people living with dementia offering them a voice to describe facilitators and barriers to living well with the condition based on their own experience. Whilst this paper has illuminated public perceptions of dementia from people living with the condition across Northern Ireland, it might not be reflective of the views of people living with dementia in other parts of the UK. Whilst authors made every attempt to engage people with dementia as widely as possible across the region, the sample was only drawn from people attending Dementia NI empowerment groups. A consequence of this approach to recruitment is that this study represents the voices of people living with mild symptoms of dementia who can maintain a high-level of independence. While the views of these participants are valid, it is unlikely these views represent people living with advanced dementia. It was also apparent from the focus group interviews that some participants had been involved in local media campaigns to promote awareness about the condition.

## Conclusion

Public perception and knowledge about dementia are improving but people living with the condition still face significant challenges in their daily lives. There is substantial evidence to suggest that local communities can facilitate people with dementia to live well with their condition. On the contrary, the converse is also equally true. Improving how people think about dementia has been a priority for many years, however despite notable improvements there are still many people living with dementia that face significant barriers to living well in their local communities.

## Data Availability

The full dataset generated and analysed during the current study are not publicly available in order to maintain the privacy of the individuals interviewed during this study. De-identified data can be made available from the corresponding author on reasonable request.

## Abbreviations

ADI: Alzheimer Disease International
CMDC: Capabilities Model of Dementia Care
DEEP: Dementia Engagement and Empowerment Project
Dementia NI: Dementia Northern Ireland
JAM Card: Just A Minute Card
UK: United Kingdom
